# Supplementary material for: Māori and Pacific People’s perspectives on Group A Streptococcus vaccine development and delivery in Aotearoa, New Zealand
Source: PLOS Glob Public Health. 2026 Feb 4;6(2):e0005787. doi: 10.1371/journal.pgph.0005787 (PMC12871991; doi:10.1371/journal.pgph.0005787)
Supplement: Attachment — Submitted filename: PLOS .docx [file pgph.0005787.s002.docx]

Thank you for the opportunity to review the manuscript entitled “Māori and Pacific People’s perspectives on Group A Streptococcus vaccine development and delivery in Aotearoa, New Zealand.” This is an interesting and relevant qualitative study addressing an important gap in public health, particularly regarding community understanding and engagement in Group A Streptococcus (GAS) vaccine development and delivery among Māori and Pacific peoples. The topic is timely and valuable for guiding culturally informed strategies to reduce the burden of rheumatic fever and related diseases.

Abstract: The abstract is clear and informative; however, it should be shorter and include a summary of the key findings or results to provide readers with a concise overview of what was discovered through this study.

Introduction: The introduction provides useful background information. However, when the authors discuss the incidence and prevalence of Group A Streptococcus infection and compare it with the most affected regions globally (such as sub-Saharan Africa), it is important to support these statements with appropriate references.
I recommend including:

Manuel V., Mocumbi A.O., Zühlke L. Rheumatic Heart Disease: Global Failure in Tackling a Common Killer. CJC Open (2025). doi: [10.1016/j.cjco.2025.09.011](https://doi.org/10.1016/j.cjco.2025.09.011)

Materials and Methods: The section is generally well described and detailed. However, it is essential to clarify how the qualitative data were analyzed.

Results: The results are clearly written, but they would benefit from visual representation of key themes or findings. The authors could consider adding:

- A thematic map, conceptual framework, or figure summarizing the major themes identified;
- A table showing representative quotations or participant statements linked to each theme.

These elements would help readers visualize and understand the findings more effectively.

Discussion

The discussion is well done and aligns with the study objectives. The authors successfully connect their findings to broader public health implications.
However, they may consider highlighting more explicitly how these community insights can inform vaccine implementation strategies or policy design in Aotearoa and similar contexts.
