# Supplementary material for: Māori and Pacific People’s perspectives on Group A Streptococcus vaccine development and delivery in Aotearoa, New Zealand
Source: PLOS Glob Public Health. 2026 Feb 4;6(2):e0005787. doi: 10.1371/journal.pgph.0005787 (PMC12871991; doi:10.1371/journal.pgph.0005787)
Supplement: Attachment — Submitted filename: Response to Reviewers.docx [file pgph.0005787.s004.docx]

**Response to Reviewers**

Reviewer #1: Thank you for the opportunity to review this excellent and timely manuscript. This study is a strong example of qualitative research that addresses a critical equity issue in global health. The use of Māori and Pacific-centred approaches provides cultural depth and ensures that the findings are grounded in the lived realities of those most affected by Group A Streptococcus. The analysis is rigorous, and the insights are both original and actionable for policymakers and health professionals.

We thank you for taking the time to review our manuscript and we appreciate your positive feedback. We have addressed your comments below.

Suggestions for Minor Revisions:

Methods:
1. You mention using convenience and purposive sampling. Could you add a sentence to briefly explain the rationale behind these choices? For example, explaining that purposive sampling for stakeholders was used to ensure key professional viewpoints were captured would be helpful.

We used purposive heterogeneous sampling to capture diverse ethic representation and perceptions of vaccines. This approach also supported accessibility, reduced barriers to participation and enabled engagement with whānau in familiar trusted environments. We used purposeful sampling to ensure representation from those working directly in Māori and Pacific healthy policy and service delivery. We have added in some additional wording to explain he rationale behind us using purposive sampling.

2. In your description of the thematic analysis, you note that three researchers analyzed the data. It would be helpful to briefly state how you reached a final consensus on the themes, as this would underscore the collaborative and rigorous nature of your analytic approach.

We have expanded the description of the thematic analysis, which now provides a clearer description of the inductive thematic analysis process, including the steps taken to code, develop, compare, and validate themes, and the roles of Māori and Pacific researchers and Governance Groups in ensuring rigour and cultural integrity.

Discussion:
1. Your findings have clear relevance beyond New Zealand. You might consider adding a sentence or two that explicitly connects your core themes (such as the legacy of colonization on health system trust and the call for self-determination) to the broader global literature on Indigenous health and vaccine equity. This would help frame your important work as a key case study with lessons that resonate internationally.

Thank you for this helpful suggestion. We have now added text to the Discussion explicitly linking our findings to the wider global literature on Indigenous health, colonisation, and vaccine equity, and positioning this study as a case example with relevance beyond Aotearoa New Zealand.

2. The limitations section is concise and appropriate. To further strengthen it, you could consider briefly noting that the study was conducted within the Auckland urban area, and perspectives might differ in other settings. This is not a weakness of the study, but acknowledging it demonstrates a comprehensive view of the research context.

Thanks for making this point. We have updated the limitations section, to reflect that views in our urban setting may differ from those in other settings.

Thank you again for producing such a high-quality and impactful study. I believe it will be an important contribution to the field and look forward to seeing it published.

Thank you again for your input, we believe that your comments have helped to strengthen our manuscript further.

Reviewer #2: Thank you for the opportunity to review the manuscript entitled “Māori and Pacific People’s perspectives on Group A Streptococcus vaccine development and delivery in Aotearoa, New Zealand.”

This is an interesting and relevant qualitative study addressing an important gap in public health, particularly regarding community understanding and engagement in Group A Streptococcus (GAS) vaccine development and delivery among Māori and Pacific peoples. The topic is timely and valuable for guiding culturally informed strategies to reduce the burden of rheumatic fever and related diseases.

Thank you for taking the time to review our manuscript, we appreciate the feedback you have given. We have addressed your points below.

Abstract
The abstract is clear and informative; however, it should be shorter and include a summary of the key findings or results to provide readers with a concise overview of what was discovered through this study.

Thank you for your point. We understand the abstract length for PLOS Global Public Health is 300 words. Ours submitted abstract was 297 words. However, we have now streamlined it somewhat, down to 264 words and highlighted the key findings. Hopefully this makes it more concise and easier to follow.

Introduction
The introduction provides useful background information. However, when the authors discuss the incidence and prevalence of Group A Streptococcus infection and compare it with the most affected regions globally (such as sub-Saharan Africa), this statement should be supported by an appropriate reference. I suggest adding:
Manuel V., Mocumbi A.O., Zühlke L. Rheumatic Heart Disease: Global Failure in Tackling a Common Killer. CJC Open (2025). doi: 10.1016/j.cjco.2025.09.011

We have added this reference as suggested into the introduction.

Materials and Methods
This section is generally well described and detailed. However, it is important to clarify how the qualitative data were analyzed. The authors should specify:

Which analytical approach was used (e.g., thematic analysis, grounded theory, framework analysis);

How the coding process was conducted and whether it was manual or software-assisted;

Whether multiple researchers were involved to ensure inter-rater reliability or validation.
Clarifying these points will improve methodological transparency and reproducibility.
Thanks for these points. We used a general inductive thematic analysis to identify themes emerging directly from the data. De-identified transcripts were manually coded by the research team using a collaboratively developed framework. Three researchers independently analysed the data, meeting regularly to compare interpretations, resolve discrepancies, and ensure consistency in theme development (see lines 136-145 of the manuscript). Provisional themes were reviewed and culturally validated by Māori and Pacific Governance groups and the Scientific Advisory Board to support reliability and methodological rigor.

Results
The results are clearly written, but they would benefit from visual representation of key themes or findings. Consider adding:

A thematic map or conceptual framework summarizing the major themes identified;

A table showing representative quotations or participant statements linked to each theme.
These additions would make the findings easier to visualize and understand.

Thanks for this. A great idea. We have added a thematic diagram, which hopefully makes the interpretation visually easy to follow.

Discussion
The discussion is well done and aligns with the study objectives. The authors successfully connect their findings to broader public health implications. However, they may consider highlighting more explicitly how these community insights can inform vaccine implementation strategies or policy design in Aotearoa and similar contexts.

Thanks for the suggestion. We have added a sentence linking our findings to vaccine implementation and policy design.

General Comment
Overall, this is a well-conceived and meaningful study with valuable implications for vaccine development and community engagement among indigenous and Pacific populations. Addressing the points above — especially regarding methodological clarity and result presentation — will strengthen the manuscript and enhance its contribution to the field.

Again, we thank you for your input and time. We believe your comments have helped strengthen this manuscript.
